# Clinical characteristics and treatment outcomes of Asian patients with T-cell large granular lymphocytic Leukemia: a single-center analysis of 67 cases

**DOI:** 10.1007/s00277-023-05575-x

**Published:** 2023-12-08

**Authors:** Taekeun Park, Ja Min Byun, Dong-Yeop Shin, Youngil Koh, Junshik Hong, Sung-Soo Yoon, Yoon Hwan Chang, Inho Kim

**Affiliations:** 1 Department of Internal Medicine, Seoul National University Hospital, Seoul, Korea; 2 Department of Laboratory Medicine, Seoul National University Hospital, Seoul, Korea; 3 Department of Laboratory Medicine, Seoul National University College of Medicine, Seoul, Korea

**Keywords:** Large granular lymphocyte, T-cell large granular lymphocytic Leukemia, Treatment

## Abstract

Large granular lymphocytic (LGL) leukemia is a clonal lymphoproliferative disorder of LGLs derived from cytotoxic T lymphocytes or natural killer cells. However, the clinical features and treatment responses are still not fully understood because of the rarity of the disease. To describe and assess a cohort of patients with T-cell large granular lymphocytic leukemia (T-LGLL). Single-center, retrospective, observational study. We retrospectively collected the clinical data of patients diagnosed with T-LGLL at Seoul National University Hospital since 2006. We included 67 patients in this study. The median age at diagnosis was 60 years. Additionally, 37 patients (55%) were symptomatic, and 25 (37%) had splenomegaly; 54 patients (81%) required treatment. Cyclophosphamide (n = 35), methotrexate (n = 25), and cyclosporin A (n = 19) were used most frequently for treatment, and their overall response rates were similar: cyclophosphamide (77%), methotrexate (64%), and cyclosporin A (63%). Splenomegaly was associated with an increased response rate to first-line therapy and a decreased complete response rate. Thrombocytopenia was associated with decreased response rates to cyclophosphamide, methotrexate, cyclosporin A, and steroids. In contrast, a high LGL number (> 2000/µL) in the peripheral blood smear was associated with increased response rates to cyclophosphamide, methotrexate, cyclosporin A, and steroids. This study describes the clinical features and treatment outcomes of patients with T-LGLL, providing valuable information for clinical decision-making regarding T-LGLL treatment.

**Supplementary Information:**

The online version contains supplementary material available at 10.1007/s00277-023-05575-x.

## Introduction

Large granular lymphocytes (LGL) are large lymphocytes with azurophilic granules, comprising 10 to 15% of normal peripheral blood mononuclear cells [1]. Large granular lymphocyte leukemia (LGLL) is a clonal lymphoproliferative disorder of LGLs, derived from either cytotoxic T lymphocytes or natural killer cells (NK-LGL) [1–3].

LGLL is a rare hematological malignancy accounting for approximately 2–5% and 5–6% of chronic lymphoproliferative disorders in North America and Asia, respectively [4, 5]. Approximately 85% of LGLL cases are derived from cytotoxic T lymphocytes, and less than 10% are chronic NK-LGL leukemia cases; the remaining cases present with aggressive NK-LGL leukemia [2, 3, 6].

Patients with T-LGLL follow an indolent course, with a median overall survival of 9–10 years [7–10]. Some patients without symptoms may be followed without treatment. However, patients mainly present with cytopenia, including anemia and neutropenia; splenomegaly is also frequently observed [7–10]. Though the pathophysiology is not fully understood, signal transducer and activator of transcription 3 (*STAT3*) and *STAT5b* mutations seem to have some roles [11–13].

Patients with symptoms, such as severe neutropenia, recurrent infections due to neutropenia, symptomatic anemia with or without transfusion, recurrent bleeding associated with thrombocytopenia, or associated autoimmune conditions, should undergo treatment [2, 4, 7, 10]. Immunosuppressive therapy is the primary form of treatment. For example, oral immunosuppressive agents, such as methotrexate (MTX), cyclophosphamide (CTX), and cyclosporin A (CsA), are widely used based on retrospective evidence [11–15]. Single immunosuppressive agents with or without steroids are usually used as first-line treatment [2, 12, 13].

Because of its rarity, most T-LGLL studies have been retrospective analyses [7–10, 12]. Moreover, few studies have been conducted in Asian countries or included patients of Asian ethnicity. Thus, retrospective analyses still play an important role in elucidating the clinical features and establishing treatment strategies. Therefore, this study assessed the clinical characteristics, treatment outcomes, and prognostic factors of a single-center cohort of 67 Asian patients with T-cell large granular lymphocyte leukemia (T-LGLL).

## Methods

### Patients

All patients diagnosed with T-LGLL at Seoul National University Hospital between 2006 and 2021 were included. We collected clinical data, including patient characteristics, clinical features, laboratory findings, immunophenotype findings, bone marrow (BM) examination results, peripheral blood smear (PBS) results, molecular analyses, treatment, and the treatment response. Hematopathology specialists reviewed the BM aspirates and PBS slides.

### Diagnostic criteria

T-LGLL diagnoses were made based on the morphological analysis of peripheral blood, a BM examination (including aspirate and biopsy), clonal T-cell receptor (TCR) γ and *β* gene rearrangement tests by polymerase chain reaction, and a STAT3/STAT5b mutation test in some cases. The BM examination and PBS were performed when the patients showed typical clinical features of T-LGLL (e.g., anemia, neutropenia, lymphocytosis, and splenomegaly). The *TCRγ* and *TCRβ* gene rearrangement test was performed when an increased number of LGLs in the PBS was confirmed or BM infiltration was identified by morphologic and immunophenotypic (e.g., CD3, CD8, CD16, and CD57) analyses. Patients with typical clinical features of clonal LGL expansion were diagnosed with T-LGLL.

### Treatment responses

Based on the complete blood count (CBC) results, anemia was defined as hemoglobin (Hb) < 12 g/dL, neutropenia as an absolute neutrophil count (ANC) < 1500/µL, and thrombocytopenia as a platelet (PLT) count < 150 × 10^3^/µL. Patients with severe neutropenia (ANC < 500/µL), recurrent infections with neutropenia, symptomatic or severe anemia, thrombocytopenia, B symptoms, or associated autoimmune conditions were also treated.

MTX (10–20 mg weekly), CsA (50–100 mg bid), or CTX (50–100 mg daily) were administered orally as a first-line treatment. Treatment responses were assessed based on the CBC results after three months of treatment. There were three treatment response categories: complete response (CR), partial response (PR), and treatment failure. A hematologic CR was defined as the normalization of CBC (Hb > 12 g/dL without transfusion, ANC > 1500/µL, PLT > 150 × 10^3^/µL, and absolute lymphocyte count < 4000/µL). A partial response (PR) was defined as CBC improvements (Hb > 8 g/dL, ANC > 500/µL, PLT > 50 × 10^3^/µL, and decreasing transfusion requirements), but the response did not satisfy the CR criteria. Treatment failure was defined as a state that did not meet the above criteria after three months of treatment.

### Statistical analyses

All statistical analyses were performed using SPSS software (version 26.0, IBM Corp., Armonk, NY, USA). The overall response rates (ORRs) were defined as the proportion of CR or PR (CR + PR) after treatment. Univariate analyses of the response rates were performed using the χ^2^ test and Fisher’s exact test when appropriate. A multivariate analysis was performed using a logistic regression model. Survival analyses were performed using the Kaplan–Meier method and compared by log-rank test. A multivariate survival analysis was performed using the Cox proportional hazards model. Statistical significance was set at p ≤ 0.05.

## Results

### Patient characteristics

We identified 67 patients with T-LGLL diagnosed between 2006 and 2021; Table 1 summarizes their clinical characteristics. The median age at diagnosis was 56 years (range, 16–80 years), but 17 patients (25%) were diagnosed before age 50. No sex-related differences were observed. Of 67 patients, 37 (55%) were symptomatic; 25 (37%) had splenomegaly, but hepatomegaly (10%), lymphadenopathy (7%), and B symptoms (4%) were rarely observed upon diagnosis.

**Table 1:** Patient characteristics

Characteristic	Number (%) (N=67)
Median age at diagnosis(range), years	56(16-80)
*Sex*	
Female	34(51)
Male	33(49)
Symptomatic	37(55)
Splenomegaly	25(37)
Hepatomegaly	7(10)
Lymphadenopathy	5(7)
B symptoms	3(4)
*Hematologic manifestation*	
Anemia	49(73)
RBC transfusion dependent	19(28)
Neutropenia (<1500/μL)	51(76)
Severe Neutropenia (<500/μL)	14(21)
Thrombocytopenia	19(28)
Lymphocytosis	47(70)
Increased LGL number in PBS (>2000/μL)	24/56(43)
Increased LGL proportion in PBS (>50%)	29/56(52)
Positive rheumatoid factor	4/22(18)
Positive antinuclear antibodies	10/31(32)
*Hematological conditions*	
PRCA	9(13)
Aplastic anemia	2(3)
Monoclonal gammopathy	2(3)
Other autoimmune diseases	3(4)
*Other malignancies*	
Hematologic	3(4)
Solid	7(10)
Needed treatment	54(81)
Lines of therapy, median (range)	2(1-7)

At the time of presentation, 49 patients (73%) had anemia (Hb < 12 g/dL), and 19 (28%) required a red blood cell transfusion. Furthermore, 51 patients (76%) had neutropenia (ANC < 1500/µL), and 14 (21%) had severe neutropenia (ANC < 500/µL). Nineteen patients (28%) presented with thrombocytopenia (PLT < 150 × 10^3^/µL), and 47 (70%) showed lymphocytosis (absolute lymphocyte count > 4000/µL).

**Fig. 1:**
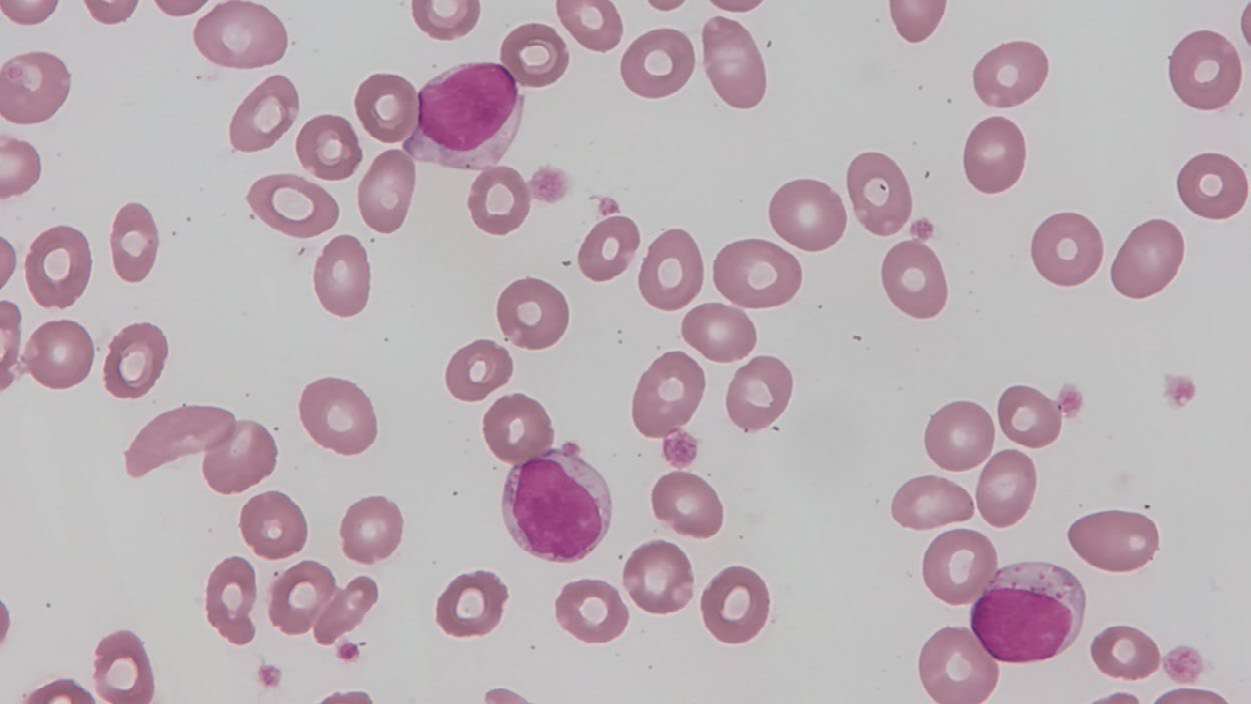
The large granular lymphocytes in the peripheral blood show moderate to abundant cytoplasm and some azurophilic granules (Wright-Giemsa stain, x1,000)

We also reviewed the PBS slides of 56 patients collected at the time of diagnosis. Only 24 patients (43%) had an increased LGL number (LGL > 2000/µL), and 29 (52%) showed an increased proportion (LGL ≥ 50%) of peripheral blood leukocytes (Fig. 1).

The serum rheumatoid factor (RF) and antinuclear antibodies (ANA) positivity rates were 18% and 32%, respectively. Three patients had uncommon autoimmune diseases (antiphospholipid syndrome, Behçet’s disease, and autoimmune encephalopathy). Regarding hematological conditions, 9 patients (13%) were diagnosed with pure red cell aplasia (PRCA), 2 (3%) with aplastic anemia, and 2 (3%) with monoclonal gammopathy of undetermined significance. Three patients (4%) had hematologic malignancies, and 7 (10%) had solid organ malignancies.

### Treatment outcomes

Overall, 54 of 67 patients (81%) required treatment. Table 2 presents the treatment responses. MTX, CTX, CsA, and steroids were the most commonly used first-line treatments; 18 patients received MTX, with a 56% ORR and 28% CR rate. Furthermore, 25 patients received CTX, with an 80% ORR and a 52% CR rate, and 5 patients received CsA, with a 40% ORR and no CRs. Three patients received steroid-only therapy as first-line treatment, with a 67% ORR and 33% CR rate. Among the remaining patients, two received danazol, one underwent splenectomy, and one received combination chemotherapy. MTX, CTX, and CsA were also commonly used as salvage treatments. Some patients did not respond to the commonly used immunosuppressive agents and required other salvage treatments, receiving azathioprine, tacrolimus, mycophenolate, danazol, or alemtuzumab.

**Table 2:** Treatment responses

Treatment	First-line			Subsequent lines			All lines		
	N.	ORR (%)	CR (%)	N.	ORR (%)	CR (%)	N.	ORR (%)	CR (%)
Methotrexate	18	10 (56)	5 (28)	7	6 (86)	1 (14)	25	16 (64)	6 (24)
Cyclophosphamide	25	20 (80)	13 (52)	10	7 (70)	2 (20)	35	27 (77)	15 (43)
Cyclosporin A	5	2 (40)	0 (0)	14	10 (71)	4 (29)	19	12 (63)	4 (21)
Steroids	3	2 (67)	1 (33)	1	0 (0)	0 (0)	4	2 (50)	1 (25)
Alemtuzumab	-	-	-	1	0 (0)	0 (0)	1	0 (0)	0 (0)
Splenectomy	1	0 (0)	0 (0)	-	-	-	1	0 (0)	0 (0)
Tacrolimus	-	-	-	4	3 (75)	0 (0)	4	3 (75)	0 (0)
Azathioprine	-	-	-	5	1 (20)	0 (0)	5	1 (20)	0 (0)
Mycophenolate	-	-	-	2	2 (100)	0 (0)	2	2 (100)	0 (0)
Danazol	2	2 (100)	1 (50)	2	0 (0)	0 (0)	4	2 (50)	1 (25)
Other	1	1 (100)	0 (0)	-	-	-	1	1 (100)	0 (0)

The ORRs for MTX, CTX, CsA, and steroids were 64%, 77%, 63%, and 50%, respectively; the CR rates were 24%, 43%, 21%, and 25%, respectively. Among the patients who received uncommon treatments, only one patient who received danazol achieved CR.

In the univariate analyses, thrombocytopenia was associated with decreased ORRs with MTX, CTX, CsA, and steroids (p = 0.014). An increased LGL number in PBS (> 2000/µL) was associated with increased ORRs with MTX, CTX, CsA, and steroids (p = 0.032). Also, an increased LGL proportion in the PBS (LGL ≥ 50%) was associated with increased ORRs with MTX, CTX, CsA, and steroids, although the results were statistically insignificant (odds ratio 8.000, p = 0.074). We also analyzed the initial response rate with various factors. Splenomegaly was associated with an increased ORR for first-line treatment (p = 0.002) and decreased CR rates (p = 0.034) for all lines of treatment. Age (> 50 or < 50 years), sex (male or female), neutropenia, severe neutropenia, anemia, the presence of PRCA, T-cell subtype (αβ or γδ), the predominancy of CD56, CD4 or CD8, and the presence of *STAT 3/5* mutation did not differ among the treatment responses (Supplement 1). In the survival analyses, none of the patient characteristics were associated with overall survival (Supplement 3).

## Discussion

Only a few prospective clinical trials of patients with LGLL have been conducted owing to the low prevalence of the disease. Consequently, a randomized phase II clinical trial for LGLL has not yet been performed [8, 16]. For this reason, many clinicians still rely on retrospective studies and empirical experience when deciding whether treatment is needed and the treatment options [2, 11–14]. Therefore, retrospective reviews are still important for clinicians until clinical trials establish sufficient evidence. Thus, we report a series of 67 patients with T-LGLL from a single center, which is the first series reported from South Korea.

We compared the clinical features of our patients with those of other more extensive retrospective studies from Western countries: one from the French registry (229 patients) [7], one from the Cleveland Clinic (204 patients) [9], and one from the Moffitt Cancer Center (319 patients) [8]. The demographic features, including age and sex, were similar between the groups. In our study, 73% and 76% of patients presented with anemia and neutropenia, respectively, much higher than those in other studies, reporting approximately 40% for each. In contrast, patients were less symptomatic in our study (55% vs. 70–80%). However, the thrombocytopenia, splenomegaly, hepatomegaly, and lymphadenopathy rates in this study were similar to those in other studies. Finally, more patients required treatment in our study than in the others. In summary, patients with T-LGLL at our center were less symptomatic but more likely to present with anemia and neutropenia, resulting in treatment. However, when we compared our study with a retrospective study from China (108 patients) [10], we found that most clinical features were similar.

Although the pathogenesis of T-LGLL is still not fully understood, there is a hypothesis that clonal LGL proliferation is caused by antigenic stimuli provided by autoantigens [2, 12, 13]. In other studies, associations between T-LGLL and autoimmune diseases, such as rheumatoid arthritis, Felty syndrome, autoimmune cytopenia, and inflammatory bowel disease, have been suggested [8]. In our study, however, only three patients (4%) presented with associated autoimmune diseases, much lower than the studies from Western countries but similar to the Chinese study. In addition, the serum RF and ANA positivity rates were 18% and 32%, respectively. Finally, T-LGLL can cause PRCA, resulting from BM suppression by cytotoxic T-LGLs; in this study, 9 patients (13%) were diagnosed with PRCA.

We also compared our cohort’s treatment responses with those of the other studies (Table 3). In our study, 54 patients required treatment, and the ORR for CTX was 77%, similar to the results from the French registry (66%) [7], Moffitt Cancer Center (62%) [8], and the phase II trial by the Eastern Cooperative Oncology Group (ECOG) (64%) [16]. The ORR for MTX was 64%, similar to that of the French registry (55%) and Moffitt Cancer Center (65%) but higher than that of the Cleveland Clinic (43%) and the phase II trial by the ECOG (38%). Finally, the ORR for CsA was 63%, similar to that of the Moffitt Cancer Center (74%) and higher than that reported in the other studies.

**Table 3:** Comparison of treatment response with other studies

Study	Total N.	Cyclophosphamide		Methotrexate		Cyclosporin A	
		N.	ORR (%)	N.	ORR (%)	N.	ORR (%)
Park et al. (2023, our study)	54	35	27 (77%)	25	16 (64%)	19	12 (63%)
French Registry (2010) [7]	100	32	21 (66%)	62	34 (55%)	24	5 (21%)
Loughran et al. (2015) [16]	59	14	9 (64%)	55	21 (38%)		
Sanikommu et al. (2018) [9]	118	53	28 (53%)	61	26 (43%)	74	36 (48%)
Dong et al. [8]	181	65	40 (62%)	89	58 (65%)	39	29 (74%)

Regarding the clinical features, it seems certain that some differences exist between Western and Asian patients. Asian patients were less symptomatic, less associated with autoimmune diseases, and had higher rates of anemia and neutropenia than their Western counteparts [10]. In contrast, treatment responses to commonly used agents were similar between the groups. Therefore, global multicenter phase clinical trials are needed for treatment evidence for T-LGLL.

Of the various patient characteristics, few have been statistically associated with treatment responses. For example, in our study, thrombocytopenia was associated with decreased ORRs with MTX, CTX, CsA, and steroids (p = 0.014, odds ratio = 0.108), and another study suggested associations between thrombocytopenia and a lower response rate and worse survival. Furthermore, splenomegaly was associated with an increased ORR after first-line treatment (p = 0.002, odds ratio = 10.000) and decreased CR rates (p = 0.034, odds ratio = 3.341) for all lines of treatment. Finally, our results suggested that an older age at diagnosis (> 50 years) may be associated with a decreased CR rate; however, the results were statistically insignificant (p = 0.057, odds ratio = 0.284, 95% confidence interval = 0.075–1.080), likely because of the small sample size (n = 54).

In the past, an increased LGL number (> 2000/µL) was mandatory for an LGLL diagnosis. However, based on recent studies, patients with LGL numbers < 2000/µL can be diagnosed with LGLL if typical clinical features are present. We had PBS slides data from 56 patients, which hematopathology specialists at our center reviewed. Of them, 24 patients (43%) had increased LGL numbers (LGL > 2000/µL), and 29 (52%) had an increased proportion of LGL (≥ 50%) among peripheral blood leukocytes. Thus, approximately half of the patients were diagnosed with LGLL without a substantial increase in the LGL number, emphasizing that clinicians should consider LGLL if other symptoms are present. In addition to its diagnostic role, an increased LGL number in PBS was associated with increased ORRs with MTX, CTX, CsA, and steroids (p = 0.032). Furthermore, an increased LGL proportion seemed to be associated with increased response rate, although the result was statistically insignificant (p = 0.074, odds ratio = 8.000, 95% confidence interval = 0.85–75.286). Therefore, our results suggest that increased LGL (in number or proportion) in PBS is an important predictive biomarker with prognostic value for treating T-LGLL.

In addition, the differences in response rates among patients with different states of LGL proliferation may be associated with different types or phases of the disease. Considering that the disease pathogenesis is not fully understood, LGLL with an increased LGL count would classify as a “proliferative type” and the other as a “cytopenic type.” From another perspective, the hypothesis that LGLL has a biphasic course of proliferative and cytopaenic phases could be considered.

In the survival analyses, none of the clinical features were associated with overall survival. According to the literature, the median overall survival of patients with T-LGLL is 9–10 years. However, the median follow-up duration in our study was 24 months, which is too short compared with other studies. Therefore, observing and assessing events (death) was difficult. Therefore, further studies are needed.

### Electronic supplementary material

Below is the link to the electronic supplementary material.

Supplementary Material 1

## Data Availability

The data of current study are available from the corresponding author on reasonable request.
