# Supplementary material for: Clinical characteristics and treatment outcomes of Asian patients with T-cell large granular lymphocytic Leukemia: a single-center analysis of 67 cases
Source: Ann Hematol. 2023 Dec 8;103(4):1235–40. doi: 10.1007/s00277-023-05575-x (PMC10940475; doi:10.1007/s00277-023-05575-x)
Supplement: Supplementary file 1 — Supplementary Material 1 [file 277_2023_5575_MOESM1_ESM.docx]

Supplement 1. Univariate comparison of response rates

Factor – ORR of CTX, MTX, CsA, steroid

| Factor | P-value | Odds ratio (95% CI) |
| --- | --- | --- |
| Sex | 1.000 | 1.105 (0.222-5.509) |
| Age | 1.000 | 1.309 (0.222-7.712) |
| Neutropenia | 1.000 | 0.545 (0.059-5.033) |
| Severe Neutropenia | 1.000 | 0.764 (0.130-4.500) |
| Anemia | 1.000 | 0.952 (0.099-9.165) |
| Severe Anemia | 0.425 | 0.466 (0.093-2.325) |
| **Thrombocytopenia** | **0.014** | **0.108 (0.018-0.643)** |
| Symptom | 0.687 | 1.453 (0.289-7.299) |
| Splenomegaly | 0.220 | 4.846 (0.540-43.462) |
| Lymphocytosis | 0.403 | 0.294 (0.033-2.652) |
| **Increased LGL number in PBS** | **0.032** | NA |
| **Increased LGL % in PBS** | **0.074** | **8.000 (0.85-75.286)** |
| RF | 1.000 | NA |
| ANA | 0.260 | NA |
| PRCA | 0.586 | NA |
| T-cell subtype (αβ or γδ) | NA | NA |
| CD56 | 1.000 | NA |
| CD4 | 1.000 | 0.875 (0.088-8.660-) |
| CD8 | 0.308 | 4.500 (0.344-58.921) |
| STAT3/5 | 0.429 | NA |

Factor – ORR of first-line therapy

| Factor | P-value | Odds ratio (95% CI) |
| --- | --- | --- |
| Sex | 0.845 | 0.891 (0.280-2.831) |
| Age | 0.740 | 1.346 (0.368-4.923) |
| Neutropenia | 1.000 | 1.000 (0.256-3.900) |
| Severe Neutropenia | 1.000 | 1.167 (0.305-4.469) |
| Anemia | 0.704 | 0.625 (0.113-3.461) |
| Severe Anemia | 0.327 | 0.565 (0.179-1.780) |
| Thrombocytopenia | 1.000 | 1.000 (0.283-3.537) |
| Symptom | 0.158 | 0.400 (0.110-1.459) |
| **Splenomegaly** | **0.002** | **10.000 (1.998-50.042)** |
| Lymphocytosis | 0.221 | 0.449 (0.123-1.643) |
| Increased LGL number in PBS | 0.745 | 1.244 (0.333-4.648) |
| Increased LGL % in PBS | 0.419 | 1.705 (0.465-6.249) |
| RF | 0.245 | NA |
| ANA | 1.000 | 1.591 (0.239-10.572) |
| PRCA | 1.000 | 1.000 (0.219-4.564) |
| T-cell subtype (αβ or γδ) | NA | NA |
| CD56 | 1.000 | NA |
| CD4 | 0.412 | 3.862 (0.436-34.247) |
| CD8 | 1.000 | 1.063 (0.090-12.598) |
| STAT3/5 | 1.000 | NA |

Factor – CR rate

| Factor | P-value | Odds ratio (95% CI) |
| --- | --- | --- |
| Sex | 0.313 | 0.570 (0.190-1.707) |
| Age | 0.057 | 0.284 (0.075-1.080) |
| Neutropenia | 0.307 | 2.000 (0.522-7.669) |
| Severe Neutropenia | 0.991 | 0.992 (0.284-3.469) |
| Anemia | 0.711 | 1.528 (0.326-7.154) |
| Severe Anemia | 0.225 | 0.504 (0.166-1.533) |
| Thrombocytopenia | 0.210 | 2.156 (0.640-7.261) |
| Symptom | 0.649 | 1.299 (0.421-4.006) |
| Splenomegaly | 0.034 | 3.341 (1.075-10.387) |
| Lymphocytosis | 0.335 | 0.571 (0.182-1.790) |
| Increased LGL number in PBS | 0.493 | 1.515 (0.461-4.981) |
| Increased LGL % in PBS | 0.841 | 1.128 (0.347-3.670) |
| RF | 0.576 | 0.250 (0.021-3.041) |
| ANA | 0.115 | 0.227 (0.050-1.299) |
| PRCA | 0.153 | 0.273 (0.051-1.461) |
| T-cell subtype (αβ or γδ) | 1.000 | 0.400 (0.016-10.017) |
| CD56 | 1.000 | NA |
| CD4 | 0.453 | 2.083 (0.443-9.790) |
| CD8 | 1.000 | 1.846 (0.157-21.690) |
| STAT3/5 | 1.000 | 2.000 (0.090-44.350) |

Supplement 2. Multivariate comparison of response rates

ORR of CTX, MTX, CsA, steroid


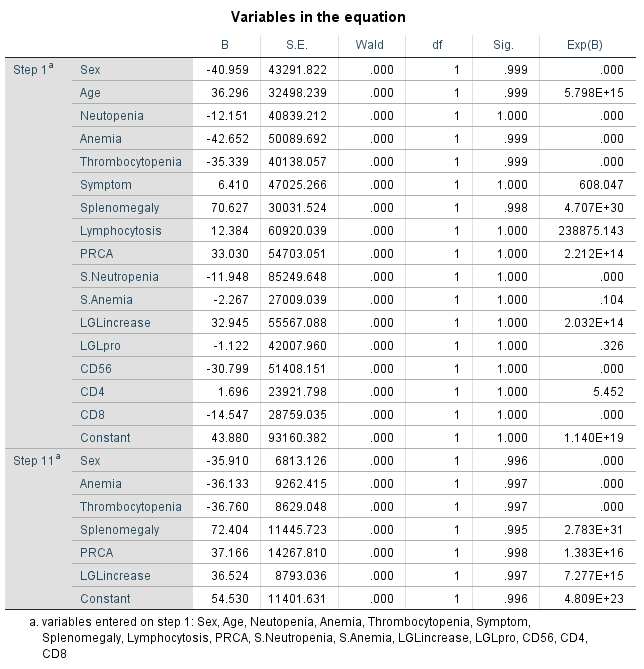


ORR of first-line therapy


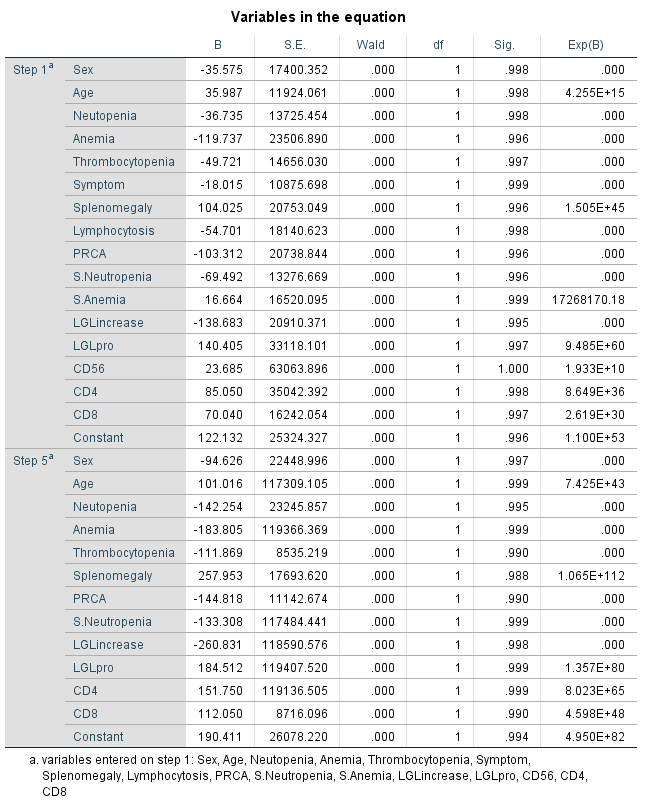


CR rate


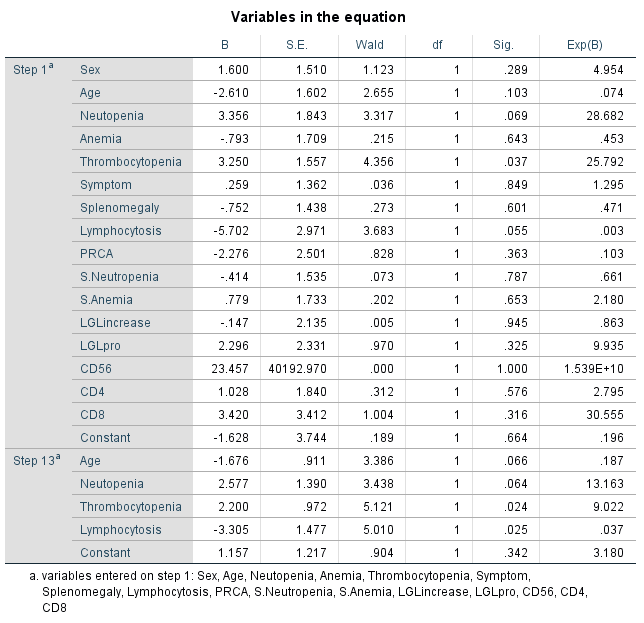


Supplement 3. Median survival (Kaplan-Meier method, compared with Log-rank test)

1. Sex p=0.164


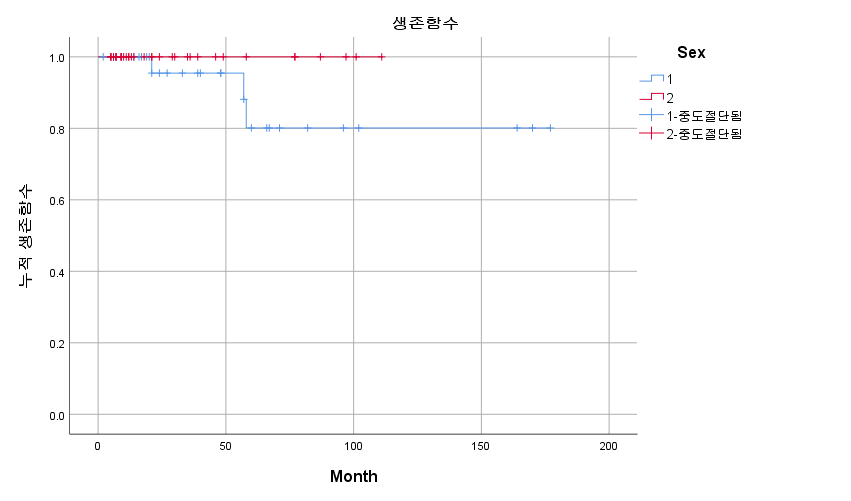


Female

Male

1. Age p=0.292

50>


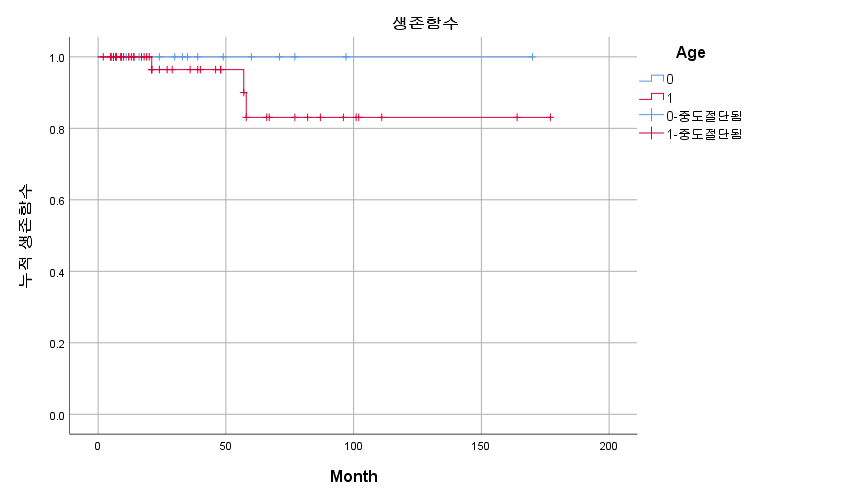


50<

1. Neutropenia p=0.323

Absent


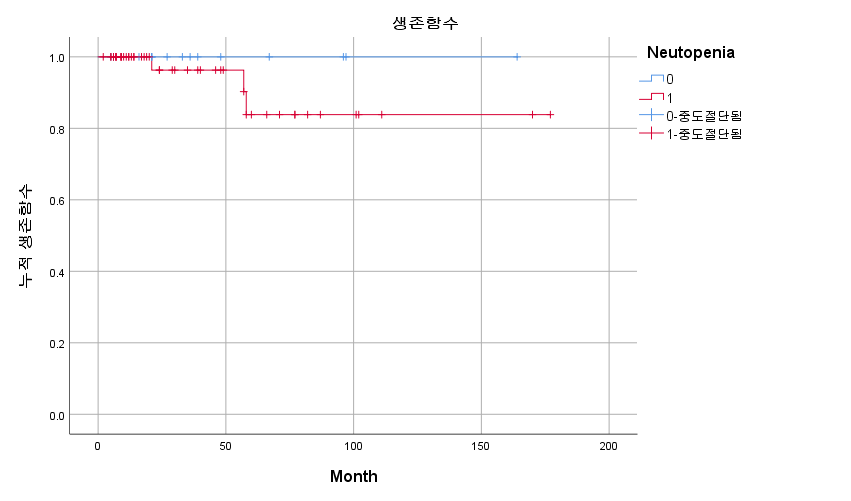


Present

1. Severe Neutropenia p=0.455


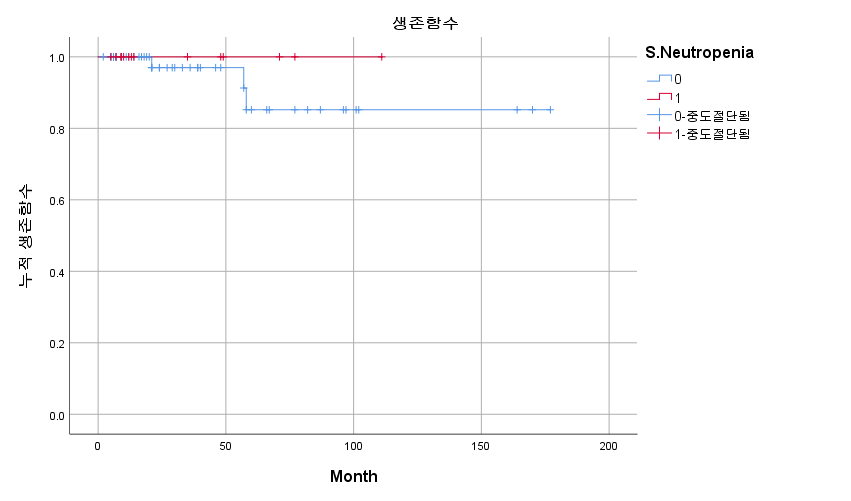


Absent

Present

1. Anemia p=0.240

Absent


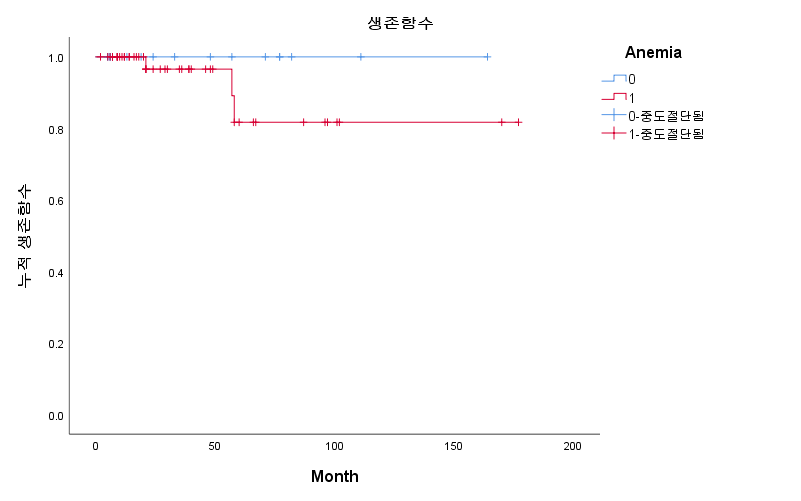


Present

1. Severe Anemia p=0.312


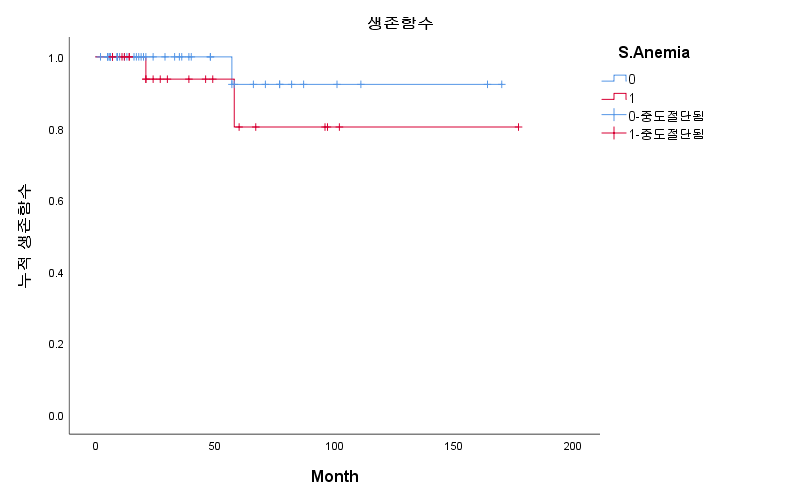


Absent

Present

1. Thrombocytopenia p=0.215

Absent


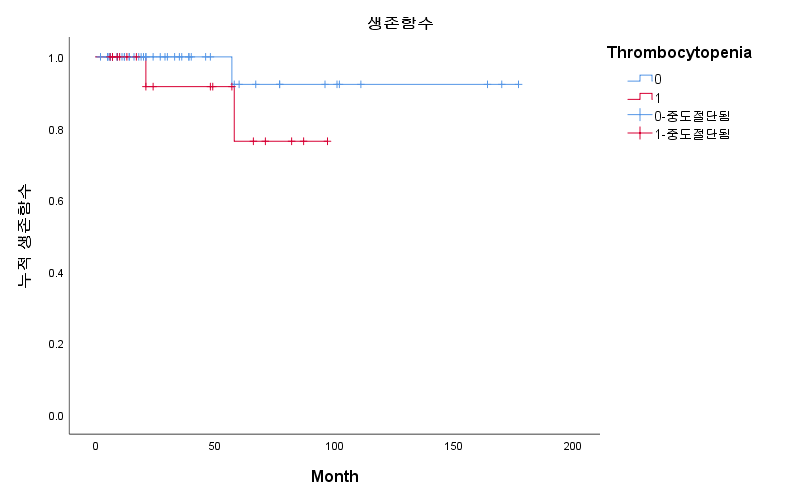


Present

1. Symptom p=0.766

Absent


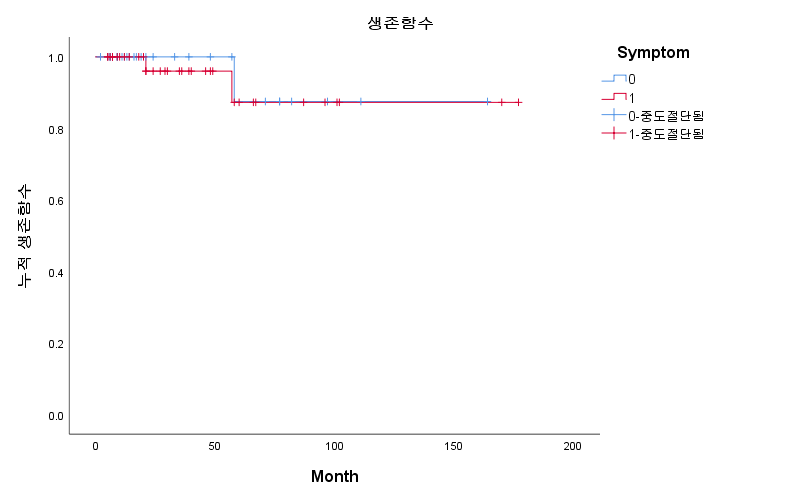


Present

1. Splenomegaly p=0.765

Present


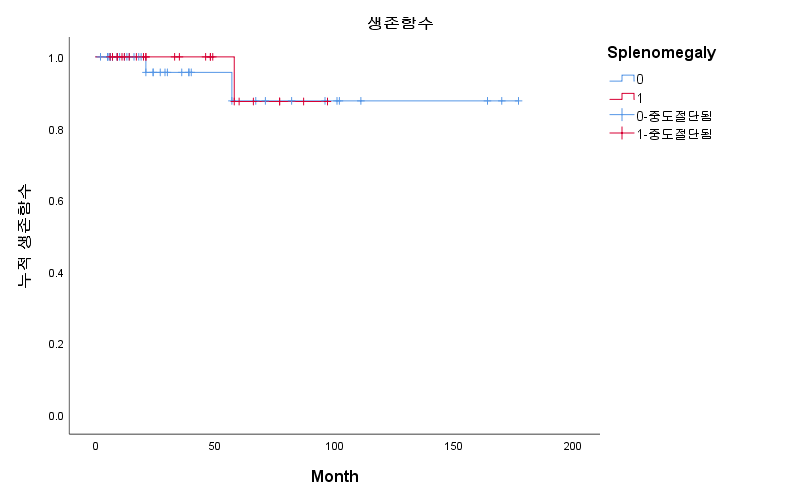


Absent

1. Lymphocytosis p=0.805


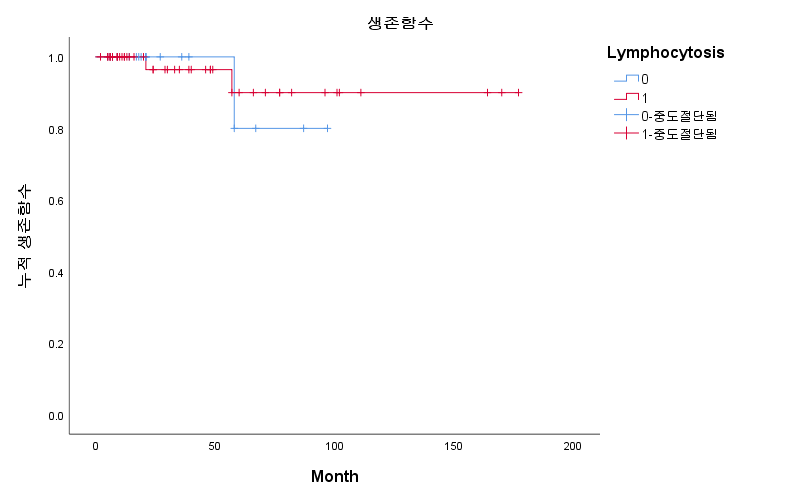


Present

Absent

1. Increased LGL in PBS p=0.526


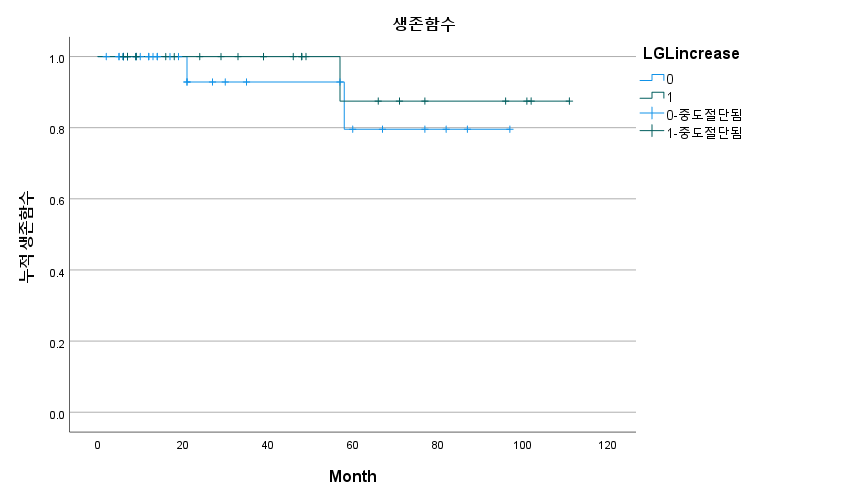


Present

Absent

1. Increased LGL % in PBS p=0.361


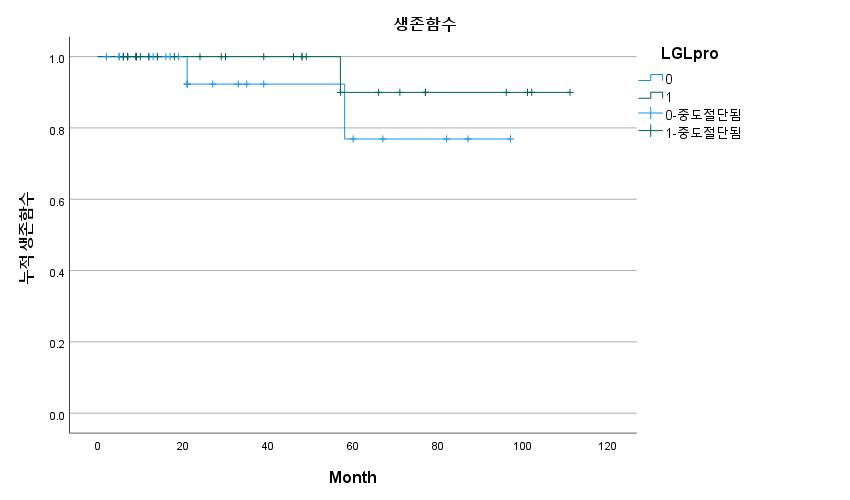


Present

Absent

1. PRCA p=0.293

Absent


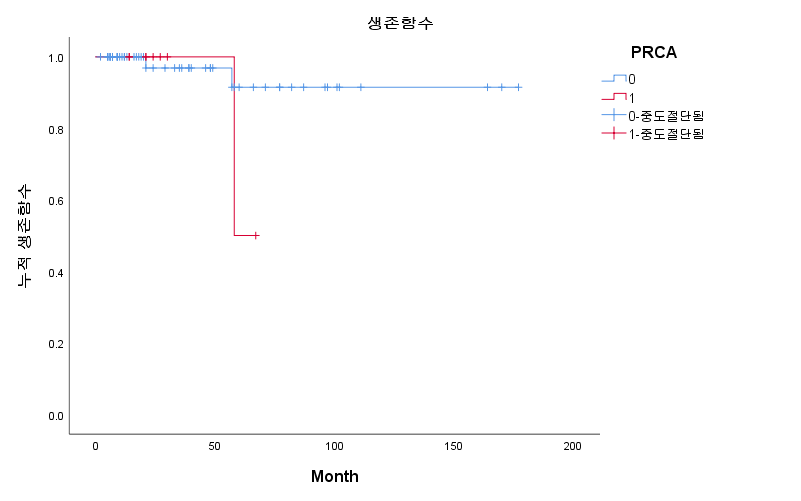


Present

1. Treatment response p<0.01


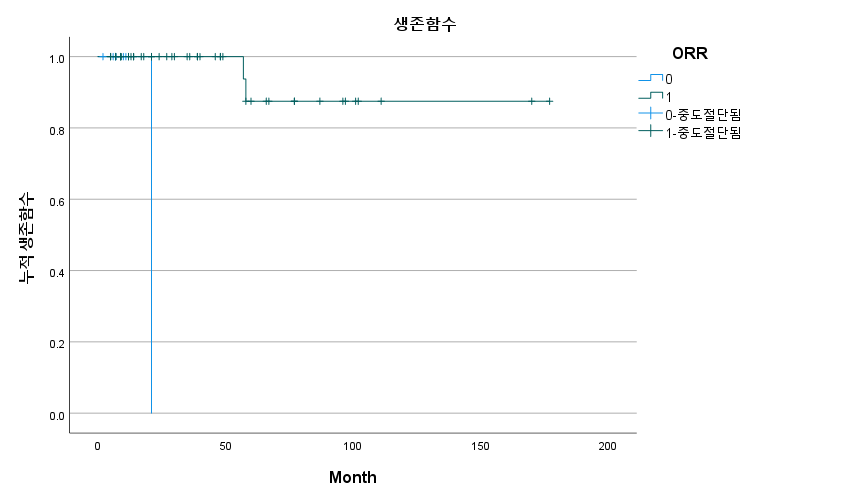


Absent

Present

1. Treatment response in first-line treatment p=0.039

Present


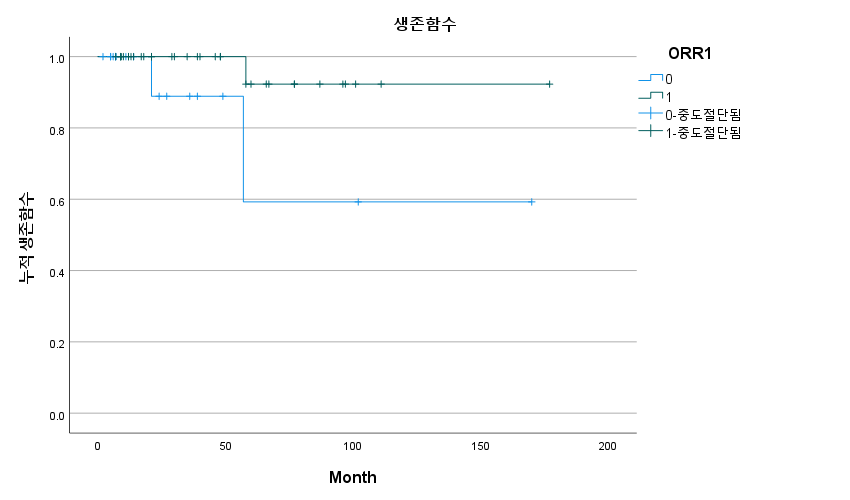


Absent

1. Complete response p=0.029


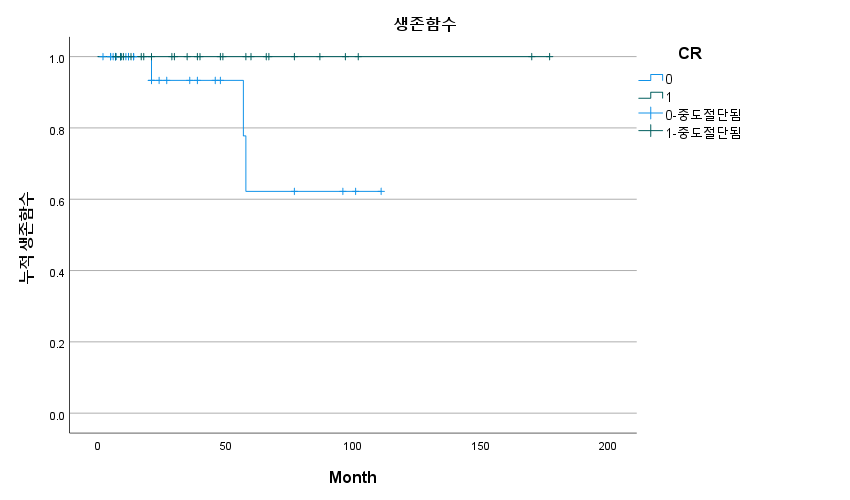


Absent

Present
